# Draft Genome Sequences of Two Enteroinvasive Escherichia coli Strains Representative of Major Enteroinvasive *E. coli* Clades

**DOI:** 10.1128/MRA.00319-21

**Published:** 2021-06-10

**Authors:** Michael J. Sikorski, Tracy H. Hazen, Gopi Vyas, Jane M. Michalski, David A. Rasko

**Affiliations:** a Institute for Genome Sciences, University of Maryland School of Medicine, Baltimore, Maryland, USA; b Department of Microbiology and Immunology, University of Maryland School of Medicine, Baltimore, Maryland, USA; c Center for Vaccine Development and Global Health, University of Maryland School of Medicine, Baltimore, Maryland, USA; University of Rochester School of Medicine and Dentistry

## Abstract

There are six described pathotypes of Escherichia coli that cause significant clinical illness in humans. Enteroinvasive E. coli (EIEC) strains have been shown to be separated into three phylogenomic clades. To add to a limited body of EIEC genomic data, we report two high-quality draft genome sequences representing different EIEC phylogenomic clades.

## ANNOUNCEMENT

Dysentery is a diarrheal disease that is most commonly caused by the bacterial genus *Shigella*; however, enteroinvasive Escherichia coli (EIEC) strains possess a pathogenic mechanism similar to that of *Shigella* species and represent an often-overlooked cause of dysentery (1, 2). The purpose of this submission is to sequence and to analyze draft genomes for two reference EIEC isolates that were previously attributed to phylogenomic clades identified by Hazen et al. (3). This will complement the previously sequenced EIEC reference isolate 53638 (4).

Isolates were obtained from human fecal matter as described previously (3) and were grown overnight at 37°C in lysogeny broth with aeration for genomic DNA preparation. Genomic DNA was purified by alkaline lysis extraction as described previously (5), with the exception that, after the phenol/chloroform extraction step, the upper aqueous phase was added to a Phase Lock Gel Heavy tube (5-Prime Inc., Gaithersburg, MD) and the extraction was repeated using chloroform/isoamyl alcohol (24:1 [vol/vol]). The upper aqueous phase was collected and at least 5 volumes of isopropanol were used for precipitation of DNA on ice for 15 min, followed by centrifugation at 12,000 × *g* for 10 min, ethanol washes, and resuspension in water. Illumina library preparation and sequencing were performed as described previously with 150-bp, paired-end reads generated for assembly error correction (6). The same genomic DNA preparations for each isolate were used to generate a sequencing library of approximately 20 kb in length and were sequenced using the Pacific Biosciences (PacBio) RS II platform with P6C4 chemistry in a single flow cell using standard methods (7). The PacBio raw data for EIEC isolates ATM460 and ATM463 were assessed for quality scores, error corrected, and assembled using the Hierarchical Genome Assembly Process (HGAP) v.3 in single-molecule real-time (SMRT) Analysis v.2.3.0 (8). Contigs were circularized, where possible, with Minimus2 (9) and were polished with the Illumina reads using Quiver (8). Contig overlaps were manually inspected and trimmed where identified. The genomes were annotated with PGAP v.4.12 (10). All software was run with default values unless otherwise specified.

Relevant statistics, including genome coverage with each sequencing technology, numbers of raw reads, contig counts, *N*_50_ values, read *N*_50_ values for PacBio reads, genome sizes, and GC contents for each genome assembly, are included in Table 1. The ATM460 assembly contains four noncircular contig fragments ranging in length from 1.3 kb to 4.7 Mb and one 279-kb circular contig. The ATM463 assembly contains six noncircular contig fragments ranging in length from 10.8 kb to 4.6 Mb and five circular contigs ranging in length from 7.4 kb to 200 kb.

**TABLE 1:** Isolate information, sequencing statistics, virulence genes, and antimicrobial resistance genes

Strain	Alternate ID^*a*^	Country of origin	Serotype	PG^*b*^	EIEC clade	No. of Illumina raw reads	No. of PacBio raw reads	Mean PacBio read length (bp)	Illumina sequence coverage (×)	PacBio sequence coverage (×)	*N*_50_ (bp)	Genome size (bp)	GC content (%)	No. of contigs	Contig name	Contig length (bp)	Contig GC content (%)	Contig form	Plasmid detected	GenBank accession no.	SRA accession no. for Illumina reads	SRA accession no. for PacBio reads
ATM460	69-3363	USA (Kentucky)	O143:H26	E	1	3,135,368	15,108	6,123.6	87.4	17.2	4,678,414	5,382,908	50.49	5	ATM460_1	279,065	46.34	Circular	IncFII *Shigella* virulence plasmid	JAALAC010000001.1	SRX8173279	SRX8173280
															ATM460_2	4,678,414	50.87	Not circular	ND^*c*^	JAALAC010000002.1		
															ATM460_3	421,407	49.12	Not circular	ND	JAALAC010000003.1		
															ATM460_4	1,315	54.98	Not circular	ND	JAALAC010000004.1		
															ATM460_5	2,707	41.89	Not circular	ND	JAALAC010000005.1		
ATM463	89-3546	Bulgaria	O164:H7	B1	3	3,521,055	21,464	7,681.78	97.3	30.4	4,621,185	5,427,144	50.86	11	ATM463_1	67,024	47.22	Circular	IncX1 antimicrobial resistance plasmid	JAALAB010000001.1	SRX8173281	SRX8173282
															ATM463_2	17,882	42.77	Circular	ND	JAALAB010000002.1		
															ATM463_3	199,809	48.58	Circular	IncFII *Shigella* virulence plasmid	JAALAB010000003.1		
															ATM463_4	8,688	60.96	Circular	IncQ1 antimicrobial resistance plasmid	JAALAB010000004.1		
															ATM463_5	7,447	48.03	Circular	ND	JAALAB010000005.1		
															ATM463_6	4,621,185	51.07	Not circular	ND	JAALAB010000006.1		
															ATM463_7	333,908	50.49	Not circular	ND	JAALAB010000007.1		
															ATM463_8	120,960	49.97	Not circular	ND	JAALAB010000008.1		
															ATM363_9	16,357	47.22	Not circular	ND	JAALAB010000009.1		
															ATM463_10	23,134	53.22	Not circular	ND	JAALAB010000010.1		
															ATM463_11	10,750	50.44	Not circular	ND	JAALAB010000011.1		

a ID, identifier.

b PG, phylogenomic group.

c ND, not detected.

Plasmid incompatibility types were predicted using PlasmidFinder v.2.0.1 (11). The assemblies for ATM460 and ATM463 both contained a *Shigella* virulence plasmid with an IncFII replicon, whereas the assembly for isolate ATM463 contained two additional closed plasmids, IncX1 and IncQ1, harboring putative antimicrobial resistance genes (Table 1).

Given the paucity of EIEC reference isolates, these two genomes will serve future studies as representative references from their respective phylogenomic clades (3).

### Data availability.

All data have been released, and accession numbers are listed in Table 1.

## References

[1] Kaper JB, Nataro JP, Mobley HLT. 2004. Pathogenic *Escherichia coli*. Nat Rev Microbiol 2:123–140. doi:10.1038/nrmicro818. 15040260

[2] Croxen MA, Finlay BB. 2010. Molecular mechanisms of *Escherichia coli* pathogenicity. Nat Rev Microbiol 8:26–38. doi:10.1038/nrmicro2265. 19966814

[3] Hazen TH, Leonard SR, Lampel KA, Lacher DW, Maurelli AT, Rasko DA. 2016. Investigating the relatedness of enteroinvasive *Escherichia coli* to other *E. coli* and *Shigella* isolates by using comparative genomics. Infect Immun 84:2362–2371. doi:10.1128/IAI.00350-16. 27271741 PMC4962626

[4] Rasko DA, Rosovitz MJ, Myers GSA, Mongodin EF, Fricke WF, Gajer P, Crabtree J, Sebaihia M, Thomson NR, Chaudhuri R, Henderson IR, Sperandio V, Ravel J. 2008. The pangenome structure of *Escherichia coli:* comparative genomic analysis of *E. coli* commensal and pathogenic isolates. J Bacteriol 190:6881–6893. doi:10.1128/JB.00619-08. 18676672 PMC2566221

[5] Sambrook JF, Fritsch EF, Maniatis T. 1989. Molecular cloning: a laboratory manual, 2nd ed. Cold Spring Harbor Laboratory, Cold Spring Harbor, NY.

[6] Kania DA, Hazen TH, Hossain A, Nataro JP, Rasko DA. 2016. Genome diversity of *Shigella boydii*. Pathog Dis 74:ftw027. doi:10.1093/femspd/ftw027. 27056949 PMC5985476

[7] Hazen TH, Michalski J, Nagaraj S, Okeke IN, Rasko DA. 2017. Characterization of a large antibiotic resistance plasmid found in enteropathogenic *Escherichia coli* strain B171 and its relatedness to plasmids of diverse *E. coli* and *Shigella* strains. Antimicrob Agents Chemother 61:e00995-17. doi:10.1128/AAC.00995-17. 28674052 PMC5571317

[8] Chin C-S, Alexander DH, Marks P, Klammer AA, Drake J, Heiner C, Clum A, Copeland A, Huddleston J, Eichler EE, Turner SW, Korlach J. 2013. Nonhybrid, finished microbial genome assemblies from long-read SMRT sequencing data. Nat Methods 10:563–569. doi:10.1038/nmeth.2474. 23644548

[9] Sommer DD, Delcher AL, Salzberg SL, Pop M. 2007. Minimus: a fast, lightweight genome assembler. BMC Bioinformatics 8:64. doi:10.1186/1471-2105-8-64. 17324286 PMC1821043

[10] Tatusova T, Dicuccio M, Badretdin A, Chetvernin V, Nawrocki EP, Zaslavsky L, Lomsadze A, Pruitt KD, Borodovsky M, Ostell J. 2016. NCBI Prokaryotic Genome Annotation Pipeline. Nucleic Acids Res 44:6614–6624. doi:10.1093/nar/gkw569. 27342282 PMC5001611

[11] Carattoli A, Hasman H. 2020. PlasmidFinder and in silico pMLST: identification and typing of plasmid replicons in whole-genome sequencing (WGS). Methods Mol Biol 2075:285–294. doi:10.1007/978-1-4939-9877-7_20. 31584170

